# Supplementary material for: Analysis of ultrasonic vocalizations from mice using computer vision and machine learning
Source: eLife. 2021 Mar 31;10:e59161. doi: 10.7554/eLife.59161 (PMC8057810; doi:10.7554/eLife.59161)
Supplement: Supplementary file 1. [file elife-59161-supp1.docx]

List of parameters and performance of Ax

| Parameter | Trial 1 | Trial 2 | Trial 3 |
| --- | --- | --- | --- |
| FS | 2.50E+05 | 2.50E+05 | 2.50E+05 |
| NFFT | 64 | 64 | 32 |
| NW | 6 | 6 | 6 |
| K | 11 | 11 | 11 |
| PVAL | 0.05 | 0.5 | 0.5 |
| channels | - | - | - |
| frequency_low | 4.50E+04 | 4.50E+04 | 4.50E+04 |
| frequency_high | 1.20E+05 | 1.20E+05 | 1.20E+05 |
| convolution_size | [1300, 0.001] | [1300, 0.001] | [1300, 0.001] |
| minimum_object_area | 18.75 | 18.75 | 18.75 |
| merge_harmonics | 1 | 1 | 1 |
| merge_harmonics_overlap | 0.9 | 0.9 | 0.9 |
| merge_harmonics_ratio | 0.1 | 0.1 | 0.1 |
| merge_harmonics_fraction | 0.9 | 0.9 | 0.9 |
| minimum_vocalization_length | 0 | 0 | 0 |
| Missed rate (%) | 37.1 | 22.34 | 4.99 |
| False discovery (%) | 45.25 | 37.67 | 57.31 |
