# Supplementary material for: Analysis of ultrasonic vocalizations from mice using computer vision and machine learning
Source: eLife. 2021 Mar 31;10:e59161. doi: 10.7554/eLife.59161 (PMC8057810; doi:10.7554/eLife.59161)
Supplement: Supplementary file 2. [file elife-59161-supp2.docx]

List of parameters and performance for MUPET

| Parameter | Trial 1 | Trial 2 | Trial 3 | Trial 4 | Trial 5 | Trial 6 | Trial 7 | Trial 8 |
| --- | --- | --- | --- | --- | --- | --- | --- | --- |
| noise-reduction | 5 | 5 | 5 | 1 | 1 | 0.5 | 2 | 1 |
| min-syllable-duration | 2 | 2 | 2 | 2 | 2 | 2 | 2 | 2 |
| max-syllable-duration | 200 | 200 | 200 | 200 | 200 | 200 | 200 | 200 |
| min-syllable-total-energy | -15 | -15 | -25 | -25 | -10 | -25 | -25 | -35 |
| min-syllable-peak-amplitude | -25 | -25 | -35 | -35 | -16 | -35 | -35 | -45 |
| min-syllable-distance | 5 | 10 | 10 | 10 | 10 | 10 | 10 | 10 |
| Missed rate (%) | 41.84 | 44.92 | 44.19 | 34.63 | 41.05 | 33.74 | 37.72 | 34.63 |
| False discovery (%) | 38.78 | 40.02 | 41.92 | 52.74 | 51.08 | 53.11 | 51.07 | 53.07 |
