# Supplementary material for: Analysis of ultrasonic vocalizations from mice using computer vision and machine learning
Source: eLife. 2021 Mar 31;10:e59161. doi: 10.7554/eLife.59161 (PMC8057810; doi:10.7554/eLife.59161)
Supplement: Supplementary file 3. [file elife-59161-supp3.docx]

List of parameters and performance for USVSEG

| Parameter | Trial 1 | Trial 2 | Trial 3 | Trial 4 | Trial 5 | Trial 6 | Trial 7 |
| --- | --- | --- | --- | --- | --- | --- | --- |
| time step | 0.5 | 0.5 | 0.5 | 0.5 | 0.5 | 0.5 | 0.5 |
| freq min | 45 | 45 | 45 | 45 | 45 | 45 | 45 |
| freq max | 125 | 125 | 125 | 125 | 125 | 125 | 125 |
| threshold | 1.5 | 2 | 2.5 | 3 | 3.5 | 4.5 | 2.5 |
| dur min | 5 | 5 | 5 | 5 | 5 | 5 | 3 |
| dur max | 300 | 300 | 300 | 300 | 300 | 300 | 300 |
| gap min | 10 | 10 | 10 | 10 | 10 | 10 | 10 |
| margin | 20 | 20 | 20 | 20 | 20 | 20 | 20 |
| read size | 15 | 15 | 15 | 15 | 15 | 15 | 15 |
| Missed rate (%) | 98.11 | 4.38 | 4.07 | 6.26 | 7.52 | 10.6 | 1.25 |
| False discovery (%) | 99.3 | 83 | 3.77 | 2.28 | 2.31 | 3.06 | 17.53 |
