# Supplementary material for: Analysis of ultrasonic vocalizations from mice using computer vision and machine learning
Source: eLife. 2021 Mar 31;10:e59161. doi: 10.7554/eLife.59161 (PMC8057810; doi:10.7554/eLife.59161)
Supplement: Supplementary file 4. [file elife-59161-supp4.docx]

List of parameters and performance for DeepSqueak

| Parameter | Value |
| --- | --- |
| overlap | 0.1 |
| frequency cut off high | 120 |
| frequency cut off low | 45 |
| neural network | MouseCall_Network_V2 |
| detection | normal |
| Missed rate (%) | 27.13 |
| False discovery (%) | 7.61 |
