# Supplementary material for: Analysis of ultrasonic vocalizations from mice using computer vision and machine learning
Source: eLife. 2021 Mar 31;10:e59161. doi: 10.7554/eLife.59161 (PMC8057810; doi:10.7554/eLife.59161)
Supplement: Supplementary file 5. [file elife-59161-supp5.docx]

VocalMat accuracy per class

| Type | N | Mean ± SEM (%) | Median [95% CI] (%) |
| --- | --- | --- | --- |
| Step up | 902 | 83.58 ± 6.50 | 91.56 [66.85, 100.00] |
| Chevron | 758 | 85.37 ± 3.93 | 85.28 [75.25, 85.48] |
| Two steps | 579 | 74.41 ± 4.16 | 70.47 [63.71, 85.11] |
| Down-FM | 557 | 90.74 ± 1.23 | 90.83 [87.56, 93.91] |
| Up-FM | 485 | 88.04 ± 2.38 | 87.59 [81.90, 94.17] |
| Short | 358 | 88.28 ± 1.88 | 89.62 [83.45, 93.11] |
| Complex | 281 | 76.64 ± 3.72 | 76.24 [67.07, 86.22] |
| Flat | 190 | 84.20 ± 4.14 | 83.51 [73.56, 94.84] |
| Step down | 142 | 84.74 ± 4.60 | 83.77 [72.90, 96.58] |
| Mult. steps | 80 | 45.89 ± 10.70 | 38.10 [16.18, 75.61] |
| Rev. Chevron | 61 | 65.18 ± 14.17 | 73.87 [28.74, 100.00] |
| Noise | 511 | 96.67 ± 0.55 | 96.67 [95.23, 98.10] |
