# Supplementary material for: Analysis of ultrasonic vocalizations from mice using computer vision and machine learning
Source: eLife. 2021 Mar 31;10:e59161. doi: 10.7554/eLife.59161 (PMC8057810; doi:10.7554/eLife.59161)
Supplement: Supplementary file 6. [file elife-59161-supp6.docx]

VocalMat accuracy considering the two most likely labels

| Type | N | Mean ± SEM (%) | Median [95% CI] (%) |
| --- | --- | --- | --- |
| Step up | 902 | 91.64 ± 4.86 | 97.18 [79.15, 100.00] |
| Chevron | 758 | 96.08 ± 1.36 | 97.20 [92.57, 99.58] |
| Two steps | 579 | 91.43 ± 1.80 | 91.77 [86.79, 96.06] |
| Down-FM | 557 | 97.08 ± 0.98 | 96.93 [94.57, 99.59] |
| Up-FM | 485 | 96.25 ± 1.40 | 97.30 [92.66, 99.84] |
| Short | 358 | 96.53 ± 1.12 | 96.72 [93.66, 99.41] |
| Complex | 281 | 92.10 ± 2.30 | 91.44 [86.20, 98.00] |
| Flat | 190 | 94.21 ± 3.96 | 97.73 [84.02, 100.00] |
| Step down | 142 | 96.11 ± 1.99 | 97.96 [91.01, 100.00] |
| Mult. steps | 80 | 83.64 ± 7.33 | 85.71 [63.28, 100.00] |
| Rev. Chevron | 61 | 77.65 ± 15.75 | 91.29 [37.17, 100.00] |
| Noise | 511 | 98.00 ± 0.45 | 97.87 [96.84, 99.17] |
